# An IMRT/VMAT Technique for Nonsmall Cell Lung Cancer

**DOI:** 10.1155/2015/613060

**Published:** 2015-10-11

**Authors:** Nan Zhao, Ruijie Yang, Junjie Wang, Xile Zhang, Jinna Li

**Affiliations:** Department of Radiation Oncology, Peking University Third Hospital, Beijing 100191, China

## Abstract

The study is to investigate a Hybrid IMRT/VMAT technique which combines intensity modulated radiation therapy (IMRT) and volumetric modulated arc therapy (VMAT) for the treatment of nonsmall cell lung cancer (NSCLC). Two partial arcs VMAT, 5-field IMRT, and hybrid plans were created for 15 patients with NSCLC. The hybrid plans were combination of 2 partial arcs VMAT and 5-field IMRT. The dose distribution of planning target volume (PTV) and organs at risk (OARs) for hybrid technique was compared with IMRT and VMAT. The monitor units (MUs) and treatment delivery time were also evaluated. Hybrid technique significantly improved the target conformity and homogeneity compared with IMRT and VMAT. The mean delivery time of IMRT, VMAT, and hybrid plans was 280 s, 114 s, and 327 s, respectively. The mean MUs needed for IMRT, VMAT, and hybrid plans were 933, 512, and 737, respectively. Hybrid technique reduced *V*_5_, *V*_10_, *V*_30_, and MLD of normal lung compared with VMAT and spared the OARs better with fewer MUs with the cost of a little higher *V*_5_, *V*_10_, and mean lung dose (MLD) of normal lung compared with IMRT. Hybrid IMRT/VMAT can be a viable radiotherapy technique with better plan quality.

## 1. Introduction

Treatment of nonsmall cell lung cancer (NSCLC) remains one of the major challenges for radiotherapy. Three-dimensional conformal radiotherapy (3D-CRT) has proved to be a promising treatment method for NSCLC allowing higher doses to be delivered to the target by improved shaping of radiation portals and conformal avoidance of normal structures compared with the conventional radiotherapy [1]. Compared to 3D-CRT, intensity modulated radiation therapy (IMRT) further significantly improved the dose conformity and sparing of organs at risk [2]. However, the longer treatment time in IMRT could increase the discomfort of the patients during the treatment, and more MUs could increase the incidence of secondary radiation-induced cancer [3, 4]. Volumetric modulated arc therapy (VMAT) provided more conformal target coverage and better sparing of organs at risk (OARs), with shorter treatment delivery time and fewer MUs than IMRT in treating cancers of different sites [5–9]. However, a larger volume of lung receiving lower dose (*V*_5_ and *V*_10_) in VMAT has been reported [10]. Dose volume histogram parameters of *V*_5_ [11–14] and *V*_10_ [12, 14, 15] have been showed to be the predictors of the radiation pneumonitis.

The aim of this study is to investigate a radiotherapy technique we call Hybrid IMRT/VMAT for nonsmall cell lung cancer treatments. The dosimetric quality and delivery efficiency of the Hybrid IMRT/VMAT technique were evaluated by comparing with IMRT and VMAT for 15 nonsmall cell lung cancer patients.

## 2. Methods and Materials

### 2.1. Patients' Characteristics

Fifteen NSCLC patients who underwent radiotherapy from January 2012 to April 2013 in our hospital were retrospectively selected for this study.

### 2.2. Delineation of Target Volumes and Critical Structures

The patients underwent four-dimensional computed tomography (4D-CT) (Brilliance Big Bore, Philips Medical Systems, Cleveland, USA) scanning in 5 mm slice thickness, 0.5 seconds of scan time per rotation during normal breathing in supine arm-up position. The gross tumor volume (GTV) was defined as the visualization of any gross tumor and lymph nodes involved (>1 cm on CT). An internal target volume (ITV) was obtained as a union of the GTVs from all respiratory motion phases. The CTV was defined as the potential harboring microscopic disease. The PTV was created by expanding the CTV by 0.5 cm. The OARs delineated included the double lungs, normal lung, spinal cord, esophagus, and heart. We defined the double lungs minus GTV as normal lung. The spinal cord and the esophagus were contoured starting at least 2 cm above the superior extent of the PTV and continuing on every CT slice to at least 2 cm below the inferior extent of the PTV. No margins were added to the organs at risk.

### 2.3. Treatment Planning

Hybrid IMRT/VMAT, IMRT, and VMAT plans were designed for each patient. The prescribed dose to the PTV was 66 Gy in 33 fractions. The plans were normalized to cover 95% of the PTV with 100% of the prescribed dose. The optimization objectives and constraints shown in Table 1 were the same for the three techniques. Eclipse 10.0 (Varian, Palo Alto, CA) treatment planning system was used for all treatment planning, utilizing 6 MV photon beams generated from Varian Trilogy linac equipped with a 120 leaf Millennium Multileaf Collimator (MLC).

### 2.4. IMRT

The beam angles of IMRT were initially optimized by the beam angle optimization algorithm (Varian Eclipse 10.0); a set of initial optimization objectives were loaded into the treatment planning system. The number of the fields was confined to five. Some beam angles were adjusted according to the experience of the dosimetrists, if the results of the beam angle optimization did not satisfy the dosimetric criteria. The plans were iteratively optimized to obtain the optimal PTV coverage and OARs sparing. After inverse planning, the leaf sequences using sliding window technique were generated for IMRT plans.

### 2.5. VMAT

All VMAT plans were generated using 2 partial arcs. The collimator angle varied between 0° and 90° according to the shape of the target while minimizing the leakage, tongue, and groove effects. Other planning parameters were MLC motion speed 0 to 2.5 cm/s, gantry rotation speed 0.5 to 4.8 degrees/s, and dose rate 0 to 600 MU/min.

### 2.6. Hybrid IMRT/VMAT

The Hybrid IMRT/VMAT technique integrates IMRT and VMAT. The IMRT part consists of a 5-field IMRT plan (Hybrid-IMRT), which contributes half of the total prescribed dose, while the VMAT parts consist of a 2 partial arcs VMAT plan (Hybrid-VMAT) which was optimized with the IMRT plan as a base plan, to deliver the other half of the prescribed dose.

### 2.7. Dosimetric Evaluation

The dosimetric quality of the Hybrid IMRT/VMAT plans was evaluated by comparison with IMRT and VMAT. To evaluate the dose distribution of the target, we calculated the minimal dose delivered to the 98% of the target volume (*D*_98%_), the maximum dose delivered to the 2% of the target volume (*D*_2%_), the median absorbed dose delivered to the 50% of the target volume (*D*_50%_), conformation number (CN), and homogeneity index (HI) according to the ICRU report 83 [16]. All parameters were computed on the basis of the DVH. The CN was defined using the equation [11]

(1) $$\begin{array}{c} \text{CN}=\frac{{\text{TV}}_{\text{RI}}}{\text{TV}}\times \frac{{\text{TV}}_{\text{RI}}}{V_{\text{RI}}}, \end{array}$$

where CN is conformation number, TV_RI_ is target volume covered by the reference isodose, TV is target volume, and *V*_RI_ is volume of the reference isodose. The CN ranged from 0 to 1, where 1 was the ideal value. A larger CN indicated a smaller volume of the prescription dose delivered outside the PTV. The HI was defined using the equation [16]

(2) $$\begin{array}{c} \text{HI}=\frac{D_{2\%}-D_{98\%}}{D_{50\%}}. \end{array}$$

An HI of 0 indicated that the dose distribution was almost homogenous. A larger HI indicated a greater dose exceeding the prescribed dose and/or a larger volume of the target receiving too small dose. The evaluation criteria of OARs were defined basically according to RTOG 1106 protocols. *V*_5_, *V*_10_, *V*_20_, *V*_30_, and mean lung dose (MLD) values were recorded and compared for normal lung, as well as the maximum dose of the spinal cord, the mean and maximum dose of the esophagus, the *D*_2%_, *V*_40_, *V*_60_, and mean dose of the heart.

### 2.8. Treatment Delivery Time and MUs

The Hybrid IMRT/VMAT, IMRT, and VMAT plans for 15 patients were delivered to a solid water phantom (Multicube Phantom, IBA, Germany) on the Trilogy linear accelerator. The treatment delivery time and MUs were recorded and evaluated. The treatment delivery time was defined as the time from first beam on until last beam off.

### 2.9. Dosimetric Evaluation Stratified by Target Volume

In order to investigate the target volume effect on the selection of the optimal technique, we separated the 15 patients into two groups according to the volumes of the PTVs, 8 patients with the PTV volumes smaller and 7 patients larger than the mean volume of the PTVs (416.1 cm^3^). The dose distribution of planning target volume (PTV) and organs at risk (OARs) for Hybrid IMRT/VMAT was compared with IMRT and VMAT for two groups separately.

### 2.10. Statistical Analysis

Paired two tailed *t*-test was used to compare the three techniques. Statistical analysis was performed using the SPSS (version 13.0, Chicago, IL) for Windows. Differences were reported to be statistically significant at *p* < 0.05.

## 3. Results

**Figure 1:**
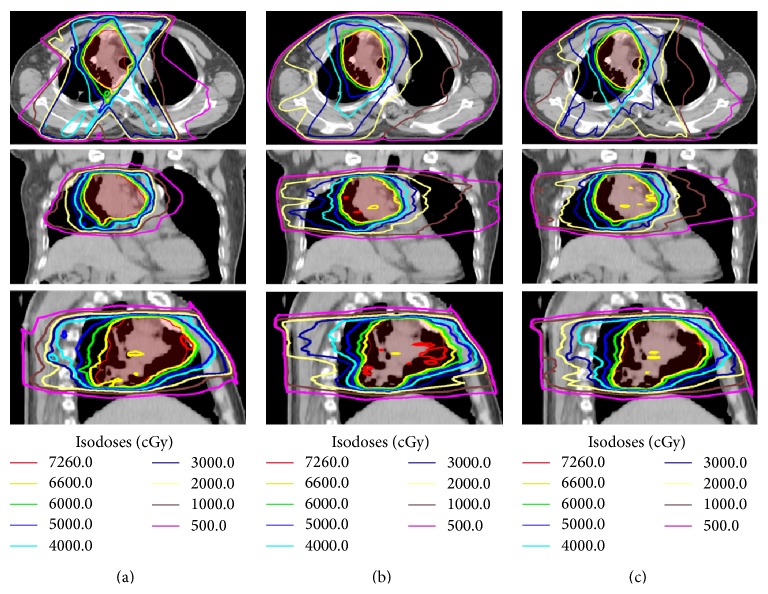
Representative axial, coronal, and sagittal computed tomography slices showing isodose distribution for (a) IMRT, (b) VMAT, and (c) Hybrid IMRT/VMAT. Planning target volume (PTV) shown in red.

**Figure 2:**
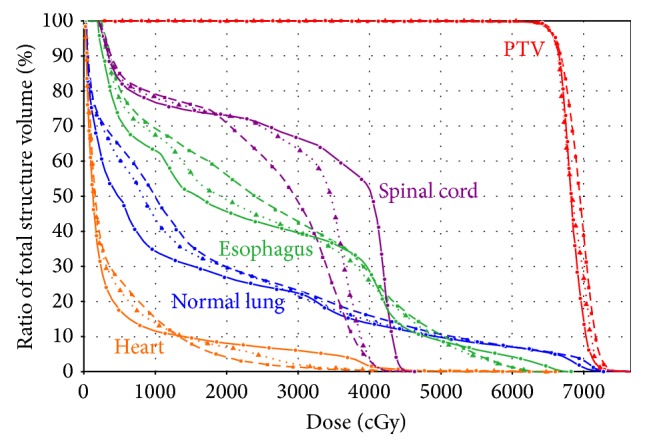
Representative dose volume histogram for IMRT, VMAT, and Hybrid IMRT/VMAT. The curves of IMRT, VMAT, and Hybrid IMRT/VMAT are indicated in solid lines, dashed lines, and dotted lines, respectively.

The mean volume of the PTV was 416.1 cm^3^ (173.4 cm^3^ to 887.0 cm^3^). For all 15 cases, all the plans were clinically acceptable in terms of target coverage, with at least 98% PTV receiving 95% of the prescribed dose. The typical isodose distribution and DVH comparison were given in Figures 1 and 2 for a patient with stage IIIB nonsmall cell lung cancer. The PTV was 414.0 cm^3^. The lesions were located in the right hilus pulmonis and the upper lobe of the right lung. The beams obtained by the beam angle optimization for IMRT are 39°, 150°, 210°, 306°, and 342°. Two partial arcs of 0° ~ 181° and 181° ~ 0° were used for VMAT.

### 3.1. Target Coverage

The data for PTV coverage and OARs sparing of IMRT, VMAT, and Hybrid IMRT/VMAT plans were summarized in Tables 2 and 3. Hybrid IMRT/VMAT significantly improved the target conformity compared with IMRT and VMAT. The mean CN was 0.79, 0.86, and 0.88 for IMRT, VMAT, and hybrid plans, respectively. Hybrid IMRT/VMAT also significantly improved the PTV dose homogeneity compared with IMRT (9.8 versus 11.3; *p* < 0.05) and VMAT (9.8 versus 12.6; *p* < 0.05). Compared with IMRT, VMAT also improved the dose conformity.

### 3.2. Organs at Risk Sparing

The *V*_30_ of normal lung for hybrid plans was significantly lower than IMRT plans (17.7% versus 18.7%; *p* < 0.05) and VMAT plans (17.7% versus 18.4%; *p* < 0.05). There was no significant difference in *V*_20_ of normal lung among three techniques. The *V*_5_, *V*_10_, and mean lung dose (MLD) of normal lung for hybrid plans were 12.6%, 8.6%, and 2.7% higher than those for IMRT plans, respectively (*p* < 0.05). However, the *V*_5_, *V*_10_, *V*_30_, and MLD of normal lung for hybrid plans were 5.1%, 7.7%, 3.8%, and 3.9% lower than those for VMAT plans, respectively (*p* < 0.05). The maximum doses of spinal cord and esophagus for hybrid plans were 5.6 Gy and 3.3 Gy lower than those for IMRT plans (*p* < 0.05). The mean doses of esophagus and heart for hybrid plans were 2.2% and 8.2% lower than IMRT plans (*p* < 0.05). The *V*_40_ and *V*_60_ of heart for hybrid plans were 27.3% and 28.9% lower than those for IMRT plans (*p* < 0.05).

### 3.3. Treatment Delivery Time and MUs

The mean delivery time of hybrid plans was longer than that of IMRT plans (327 s versus 280 s; *p* < 0.05) and that of VMAT plans (327 s versus 114 s; *p* < 0.05). The number of mean MUs of hybrid plans (797 ± 81) was between the values of IMRT (997 ± 140) and VMAT plans (509 ± 53).

### 3.4. Dosimetric Evaluation Stratified by Target Volume

For the patients with the PTV volume smaller than 416.1 cm^3^, the mean CN was 0.72, 0.86, and 0.89 for IMRT, VMAT, and hybrid plans, respectively. Hybrid plans also significantly improved the PTV dose homogeneity compared with IMRT (9.9 versus 17.1; *p* < 0.05) and VMAT (9.9 versus 14.9; *p* < 0.05). The mean *V*_5_ and *V*_10_ of normal lung for hybrid plans were 31.3% and 19.0%, with an absolute difference of 4.1% and 1.1% lower than those for VMAT plans (*p* < 0.05), respectively. The MLD for hybrid plans was 6.8 Gy, 0.4 Gy lower than that for VMAT plans (*p* < 0.05). No difference of *V*_20_ of normal lung among the IMRT, VMAT, and hybrid plans was found. The mean *V*_30_ of normal lung for hybrid plans was 20.3% lower than that for IMRT plans (*p* < 0.05). No significant difference was found in the mean *V*_30_ of normal lung between hybrid and VMAT plans. The maximum dose of spinal cord for hybrid plans was 27.6 Gy, which was 4.3 Gy lower than that for IMRT plans (*p* < 0.05). The mean dose of esophagus for hybrid plans was 9.4 Gy, which was 0.7 Gy lower than that for VMAT plans (*p* < 0.05). No differences in the mean *D*_max_ of esophagus and *D*_2%_, mean dose, *V*_60_, *V*_40_ of heart among the IMRT, VMAT, and hybrid plans were found.

For the patients with the PTV volume larger than 416.1 cm^3^, the mean CN was 0.64, 0.80, and 0.83 for IMRT, VMAT, and hybrid plans, respectively. Hybrid plans also significantly improved the PTV dose homogeneity compared with IMRT (10.0 versus 14.6; *p* < 0.05). The mean *V*_5_ of normal lung for hybrid plans was 49.2%, with an absolute difference of 4.4% lower than that for VMAT plans (*p* < 0.05), while no difference was found for *V*_10_ between two techniques. The MLD for hybrid plans was 10.4 Gy, 0.6 Gy lower than that for VMAT plans (*p* < 0.05). No differences of *V*_20_ and *V*_30_ of normal lung among the IMRT, VMAT, and hybrid plans were found. The maximum dose of spinal cord for hybrid plans was 37.0 Gy, which was 5.8 Gy lower than that for IMRT plans (*p* < 0.05). No differences of *D*_max_, mean dose of esophagus and *D*_2%_, mean dose, *V*_60_, *V*_40_ of heart among the IMRT, VMAT, and hybrid plans were found.

## 4. Discussion

In this study, we investigated a Hybrid IMRT/VMAT technique for primary nonsmall cell lung cancer. Compared with IMRT and VMAT, the improvements in conformity and homogeneity with Hybrid IMRT/VMAT were especially important when the target was in close proximity to the spinal cord limiting a satisfactory coverage of PTV. Compared with IMRT, Hybrid IMRT/VMAT significantly reduced the irradiated volume of the OARs and normal tissue receiving medium to high dose. Compared with VMAT, Hybrid IMRT/VMAT reduced the volume of normal lung receiving dose higher than 5 Gy, 10 Gy, 30 Gy, and MLD significantly.

Several studies suggested that *V*_5_ [11–14], *V*_10_ [12, 14, 15], and MLD [14, 15, 17, 18] were correlated with radiation pneumonitis, although the determination of the contributors to radiation pneumonitis was challenging, since a variety of treatment/patient-related factors appeared to influence this risk.

There were several studies demonstrating that VMAT could reduce delivery time and MUs compared with IMRT [12–15]. Reduction of delivery time could decrease the possibility of the intrafraction patient motion that leads to target underdosage and/or worse OARs sparing. However, the treatment delivery time of hybrid plans was longer than that of VMAT and IMRT plans in our study, because a hybrid plan comprised of both a 5-field IMRT and a 2 partial arcs VMAT. Liu et al. [19] reported that IMRT plans with fewer beams (five or seven beams) could achieve dosimetric quality comparable to those using nine equal-spaced beams, with reduced MUs and field segments. Using nine equal-spaced beams could allow more conformal plans but increased *V*_5_ and *V*_10_ of normal lung. So, we used 5-field IMRT plans to reduce the low dose distribution for normal lung. Chan et al. [20] reported that, in their pilot study of using VMAT, dosimetric distribution of one full arc was less favorable compared to those with two half arcs. So, 2 partial arcs VMAT was a good choice to compare with IMRT and Hybrid IMRT/VMAT.

Hybrid IMRT/VMAT improved the target dose conformity and homogeneity compared with IMRT and VMAT, while the difference of dose homogeneity of hybrid and VMAT plans became insignificant for the patients with the PTV volume larger than 416.1 cm^3^. The possible reason was that IMRT and VMAT made compromises in different aspects. IMRT achieved a reasonable dose distribution by intensity modulation with limited angular beam sampling. Due to the sparse angular sampling in IMRT, the conformity of the resultant dose distribution was often limited. On the other hand, while VMAT had sufficient angular sampling, it did not provide the desired intensity modulation in some beam directions. The final dose distribution depended on the level of intensity modulation and angular sampling. Hybrid IMRT/VMAT improved the target conformity and homogeneity by increasing the freedom to find the optimal combination of angular sampling and intensity modulation. The reason for the insignificance of homogeneity difference with increasing target volume between VMAT and hybrid plans was perhaps due to the fact that the homogeneity saturated by increasing the angular sampling in VMAT beyond a certain level, with the side effect of spreading low dose, which was also demonstrated as the reduced *V*_30_ in VMAT and hybrid plans compared with IMRT for smaller targets, whereas no difference was found among three techniques for larger targets.

Several recent publications have introduced hybrid techniques which consisted of IMRT and arc with the purpose of combining the efficiency of arc and OARs sparing of IMRT. Martin et al. [17] reported that a novel IMRT & Arc technique consisted of 4-field IMRT in conjunction with a conformal arc. They demonstrated that for patients with esophageal cancers the IMRT & Arc technique could potentially improve the therapeutic ratio in reduction of cardiorelated and pulmonary toxicity compared with plans for either helical tomotherapy or single-arc RapidArc plans. The forward planning for the conformal arc, as well as the manual IMRT beam arrangement, was used in their study. Similarly, Robar and Thomas [18] reported a HybridArc technique combining optimized dynamic conformal arcs and IMRT. In contrast to VMAT component in Hybrid IMRT/VMAT, the arc component of IMRT & Arc and HybridArc did not involve intensity modulation, for example, via dose rate or gantry speed modulation, overlapping multiple arcs, or associated linac functionality. Compared with Hybrid IMRT/VMAT technique, the degrees of freedom of IMRT & Arc and HybridArc were limited by (1) only a single pass by each arc, (2) constant dose rate, and (3) constant gantry speed. So no improvements in the brainstem and optic chiasm sparing were found in HybridArc compared with IMRT for the complex cranial cases. Chan et al. [20] reported that the Hybrid-RapidArc technique utilizing two arcs with additional static conformal fields could produce lower *V*_5_, *V*_10_, and MLD than double arcs RapidArc technique for lung cancers. However, Hybrid-RapidArc failed to meet the plan acceptance criteria due to the limited ability of intensity modulation with the conformal radiotherapy component, especially for the challenging cases (highly irregular PTV), with involving mediastinal lymphadenopathy. Furthermore, the ability to reduce the volume of normal lung receiving low doses was limited, because the intensity of the static beams could not be modulated to achieve good target conformity.

We developed a Hybrid IMRT/VMAT technique using IMRT as the base plan and then optimized the VMAT plan achieving trade-off between better dosimetric quality of IMRT and delivery efficiency (fewer MUs) of VMAT. This technique can be used on any treatment planning system capable of producing both VMAT and IMRT plans. Additional research work on the Hybrid IMRT/VMAT strategy is warranted in several areas. Most notable is to develop an optimization algorithm which can optimize both VMAT and IMRT simultaneously to determine the optimal proportion of the prescribed dose for the IMRT and VMAT components, the delivery sequence integrating the IMRT and VMAT components. Furthermore, the types of cancer sites and geometries that will benefit most from this Hybrid IMRT/VMAT technique should be further investigated.

We investigated the influence of prescription dose ratio between IMRT and VMAT in Hybrid IMRT/VMAT on the dose distribution and delivery efficiency, by creating the plans with the weighting of IMRT to VMAT of 1 : 1, 1 : 2, and 2 : 1. The results demonstrated that better conformity, homogeneity, sparing of normal lung from higher dose irradiation, and delivery efficiency were obtained with the increasing weight of the VMAT, with the cost of increasing the volume of low dose to normal lung (*V*_5_, *V*_10_) and MLD. In addition, the ideal number of IMRT beams and VMAT arcs and the start and stop angle of arcs in hybrid plans would likely vary for different cases. For the representative case in this study, the beam angles of the IMRT plan were optimized using the beam angle optimization algorithm. Two right-anterior oblique fields and a right-posterior oblique field with gantry angles of 342°, 306°, and 210°, a left-anterior oblique field with gantry angle of 39°, and a left-lateral field with gantry angle of 150° were used. For the VMAT plan, two half arcs with the gantry angle 181° to 0° and 0° to 181° were used. We will further investigate a feasibility of automatic determination of these parameters for the individual patients in the optimization, so that the full potential of hybrid technique can be explored and the hybrid plans can be planned and delivered together, not separately. Hoover et al. [21] investigated an optimization and delivery technique called united intensity-modulated arc therapy (UIMAT), which optimized IMRT and VMAT simultaneously and delivered IMRT and VMAT in the same arc. They found that UIMAT has the potential to be superior to IMRT or VMAT.

The Hybrid IMRT/VMAT technique can be implemented to find the optimal compromise between gantry-angle and intensity modulation degrees of freedom, dosimetric quality, and delivery efficiency. It may be delivered without switching between delivery techniques in the future. That is, hybrid plans will be delivered as modulated arcs with IMRT inside, that is, IMRT control points (with no gantry motion) within a VMAT control point sequence (with gantry changes) rather than current two separate components, so that the delivery time would be further reduced. In addition, the emergence of autofield sequencing, which eliminates the unnecessary operator manual control of gantry rotation during dose delivery, and the dramatically increased dose rate in modern digital LINACs will make Hybrid IMRT/VMAT more efficient.

Our previous study demonstrated that some gantry angles benefited plan quality the most from beam modulation for some specific targets and OARs configuration [22]. Li and Xing [23] and Matuszak et al. [24] also demonstrated that an additional modulation from “optimal” beam angle improved plan quality compared with VMAT alone. While there were some optimal beam orientations that would benefit from IMRT, the selection of the best beam orientations for modulation might become increasingly difficult for the complicated cases. Li and Xing [23] proposed a dense angularly sampled and sparse intensity modulated RT (DASSIM-RT) strategy, in which a large number of beam angles were used to increase the angular sampling while simplifying the intensity modulation by eliminating the dispensable segments, to improve dose distribution while maintaining high delivery efficiency. In contrast with Hybrid IMRT/VMAT, DASSIM-RT utilized an IMRT delivery mode, which could be time intensive. In addition, the number of beams and intensity level were arbitrarily selected in DASSIM-RT. Matuszak et al. [24] reported a similar strategy called FusionArc and proposed and validated gradient factor as the metric to find the optimal IMRT beam directions. They used a single-arc VMAT plan as the baseline plan and then converted selected VMAT apertures with the highest gradient into IMRT beams. Different from the arbitrarily selecting the number of beams and intensity level in DASSIM-RT, and using one arc and sequentially converting IMRT beam one by one in FusionArc, in our study, the Hybrid IMRT/VMAT integrated 5 IMRT fields and 2 partial VMAT arcs, in which the optimal IMRT beam directions were created by using beam angle optimization, while the VMAT arcs were optimized with the IMRT part as a base plan.

## 5. Conclusions

In combining VMAT and IMRT beams, Hybrid IMRT/VMAT significantly improved both the target dose conformity and the homogeneity compared with IMRT and VMAT for nonsmall cell lung cancer. It reduced *V*_5_, *V*_10_, *V*_30_, and MLD of normal lung compared with VMAT and protected the OARs better with fewer MUs with the cost of a little higher *V*_5_, *V*_10_, and mean lung dose (MLD) of normal lung compared with IMRT. Hybrid IMRT/VMAT technique can be a viable radiotherapy technique with better plan quality.

## Figures and Tables

**Table 1:** Treatment planning objectives used for Hybrid IMRT/VMAT, IMRT, and VMAT plans.

PTV	*D*_98%_	>62.7 Gy
	*D*_2%_	<72.6 Gy

Normal lung	^*∗*^ *V*_5_	<60%
	^*∗*^ *V*_10_	<40%
	^*∗*^ *V*_20_	<30%
	^*∗*^ *V*_30_	<20%
	Mean dose	<16 Gy

Spinal cord	Max dose	<50 Gy

Esophagus	Max dose	<66 Gy
	Mean dose	<34 Gy

Heart	^*∗*^ *V*_40_	<80%
	^*∗*^ *V*_50_	<30%
	Mean dose	<30 Gy

PTV is planning target volume.

^*∗*^ *V*_*N*_ is percentage volume of OARs receiving at least *N* Gy of radiation dose.

**Table 2:** The data for PTV coverage for IMRT, VMAT, and Hybrid IMRT/VMAT plans.

	IMRT	VMAT	Hybrid	IMRT versus VMAT	IMRT versus Hybrid	VMAT versus Hybrid
	Mean ± SD	Mean ± SD	Mean ± SD	*p* value	*p* value	*p* value
PTV						
*D*_98%_ (Gy)	64.6 ± 0.5	64.6 ± 0.5	65.0 ± 0.3	>0.05	<0.05	<0.05
*D*_2%_ (Gy)	72.6 ± 1.7	73.3 ± 1.7	71.5 ± 1.0	>0.05	<0.05	<0.05
CN	0.79 ± 0.05	0.86 ± 0.04	0.88 ± 0.03	<0.05	<0.05	<0.05
HI (%)	11.3 ± 0.7	12.6 ± 0.6	9.8 ± 0.3	<0.05	<0.05	<0.05

PTV is planning target volume, IMRT is intensity modulated radiation therapy, VMAT is volumetric modulated arc therapy, CN is conformation number, and HI is homogeneity index.

**Table 3:** The data for OARs sparing for IMRT, VMAT, and Hybrid IMRT/VMAT plans.

	IMRT	VMAT	Hybrid	IMRT versus VMAT	IMRT versus Hybrid	VMAT versus Hybrid
	Mean ± SD	Mean ± SD	Mean ± SD	*p* value	*p* value	*p* value
Normal lung						
*D*_2%_ (Gy)	67.5 ± 2.7	67.9 ± 3.7	67.7 ± 3.2	>0.05	>0.05	>0.05
^*∗*^ *V*_30_ (%)	18.7 ± 4.1	18.4 ± 4.2	17.7 ± 3.9	>0.05	<0.05	<0.05
^*∗*^ *V*_20_ (%)	25.4 ± 4.9	25.2 ± 6.1	25.5 ± 5.6	>0.05	>0.05	>0.05
^*∗*^ *V*_10_ (%)	35.2 ± 6.9	41.7 ± 8.0	38.5 ± 7.3	<0.05	<0.05	<0.05
^*∗*^ *V*_5_ (%)	50.0 ± 8.2	60.3 ± 11.2	57.2 ± 10.7	<0.05	<0.05	<0.05
Mean (Gy)	14.2 ± 2.2	15.2 ± 2.6	14.6 ± 2.3	<0.05	<0.05	<0.05
Spinal cord						
*D*_max⁡_ (Gy)	41.5 ± 10.0	35.7 ± 10.5	35.9 ± 9.0	<0.05	<0.05	>0.05
Esophagus						
*D*_max⁡_ (Gy)	67.0 ± 5.7	66.2 ± 6.9	63.7 ± 7.9	>0.05	<0.05	<0.05
Mean (Gy)	22.9 ± 9.9	23.0 ± 9.6	22.4 ± 9.7	>0.05	<0.05	<0.05
Heart						
*D*_2%_ (Gy)	34.0 ± 25.7	31.3 ± 24.0	31.6 ± 23.8	>0.05	<0.05	>0.05
Mean (Gy)	8.5 ± 8.8	7.4 ± 7.1	7.8 ± 7.8	<0.05	<0.05	>0.05
^*∗*^ *V*_60_ (%)	1.1 ± 2.0	0.9 ± 1.6	0.8 ± 1.5	<0.05	<0.05	>0.05
^*∗*^ *V*_40_ (%)	4.5 ± 5.6	2.8 ± 4.0	3.2 ± 4.4	<0.05	<0.05	>0.05

PTV is planning target volume, IMRT is intensity modulated radiation therapy, VMAT is volumetric modulated arc therapy, CN is conformation number, and HI is homogeneity index.

^*∗*^ *V*_*N*_ is percentage volume of OARs receiving at least *N* Gy of radiation dose.
